# Determinants of Physical Activity Based on the Theory of Planned Behavior in Iranian Military Staff’s Wives: A Path Analysis

**DOI:** 10.5539/gjhs.v7n3p230

**Published:** 2014-11-30

**Authors:** Zeinab Gholamnia Shirvani, Fazlollah Ghofranipour, Reza Gharakhanlou, Anoshirvan Kazemnejad

**Affiliations:** 1 Department of Health Education, Faculty of Medical Sciences, Tarbiat Modares University, Tehran, Iran; 2 Department of Physical Education & Sport, Faculty of Humanities, Tarbiat Modares University, Tehran, Iran; 3 Department of Biostatistics, Faculty of Medical Sciences, Tarbiat Modares University, Tehran, Iran

**Keywords:** path analysis, physical activity, Theory of Planned Behavior

## Abstract

Level of physical activity as a key determinant of healthy lifestyle less than is required in individuals particularly women. Applying theories of behavioral change about complex behaviors such as physical activity leads to identify effective factors and their relations. The aim of this study was to determine predictors of physical activity behavior based on the Theory of Planned Behavior in military staff’s wives in Tehran. This cross-sectional study was performed in 180 military personnel’s spouses residing in organizational houses, in Tehran, Iran in 2014. The participants were randomly selected with multi-stage cluster sampling. The validity and reliability of the theory based scale evaluated before conducting the path analysis. Statistical analysis was carried out using SPSS16 and LISREL8.8. The results indicated the model explained 77% and 17% of intention and behavior variance. Subjective norms (Beta=0.83) and intention (Beta=0.37) were the strongest predictors of intention and behavior, respectively. The instrumental and affective attitude had no significant path to intention and behavior. The direct relation of perceived behavioral control to behavior was non-significant. This research demonstrated relative importance and relationships of Theory of Planned Behavior constructs in physical activity behavior of military personnel’s spouses in Tehran. It is essential to consider these determinants in designing of educational interventions for promoting and maintaining physical activity behavior in this target group.

## 1. Introduction

Physical inactivity is a modifiable risk factor for cardiovascular disease and a widening variety of other chronic diseases, including diabetes mellitus, cancer, obesity, hypertension, bone and joint diseases and depression (Warburton, Nicol, & Bredin, 2006). Inactivity rises with age, is higher in women than men (Hallal et al., 2012). The Eastern Mediterranean Region has one of the highest rates of physically inactive people in the world. One in three men and one in two women do not follow the minimum recommended levels of physical activity (World Health Organization [WHO], 2014). Almost 50% of women and 36% of men are insufficiently active in this region (WHO, 2008). In Iran, 47% of women and 27.1% of men are insufficiently active (based on age- standardized estimate) (WHO, 2014). Health indicators show that only 31.3% of women and 43.4% of men are physically active at least 10 minutes in leisure time in Tehran (khosravi et al., 2009). Also, research priorities of a military medical sciences university point to low level of activity in military staff’s wives in Tehran.

Given the complexity of physical activity behavior, it is necessary to use behavior change theories to identify the main factors influencing the behavior and relationships between them and key elements of interventions (Nutbeam, Harris, & Wise, 2010). The Theory of Planned Behavior (TPB) (Ajzen, 1991) is one of the leading theories applied to predict a range of health behaviors, such as physical activity (Armitage, 2005; Eng & Martin Ginis, 2007; Johnston et al., 2007). TPB theorizes that behavioral intention (a conscious motivation to act) is the primary determinant of any given behavior that is influenced by attitudes toward the behavior (positive and/or negative evaluations of performance), subjective norms (perceptions of social norms to act) and perceived behavioral control (perceptions of controllability and ease of performance) (de Bruijn, Rhodes, & Van Osch, 2012). TPB has been found to typically explain 41-46% of the variance in physical activity intentions and 24-36% of the behavior variance (Hagger, Chatzisarantis, & Biddle, 2002; McEachan, Conner, Taylor, & Lawton, 2011).

To our best knowledge, no (theory-based) study had performed about exercise behavior (as a key determinant of healthy lifestyle) in military personnel’s spouses in Tehran/Iran. Although, military staffs are trained about physical fitness in accordance with job demands, their wives are inactive or less active. Mortality, morbidity and unhealthy behaviors of these women impact on health and health behaviors of military families (Similar to other families in the society). Women can play important role in forming active lifestyle in their family and community (Mohseni, 2003). Hence, to identify exercise behavior predictors will assist to conduct TPB based research for promoting and maintaining physical activity behavior in this target group. On the other hand, occupational success of military staffs in order to ensure the security and peace of a country depends on their living in healthy families (Ahmadi, Fathi Ashtiani, & Habibi, 2009). Therefore, investigating determinants of women’s exercise behavior for improving active lifestyle can help to provide a capable military force. Accordingly, current research was carried out for some reasons; (a) prevalence of inactivity and low activity in Iranian/Tehranian women, particularly military personnel’s spouses; (b) effective role of TPB to promote and maintain physical activity behavior; (c) lack of research in examining of exercise behavior; and its determinants in military staff’s wives. The purpose of this study was to identify predictors of physical activity behavior based on the TPB on military personnel’s wives in Tehran.

## 2. Methods

### 2.1 Participants and Sampling Procedures

We conducted a cross-sectional study utilizing the path analysis on military personnel’s spouses residing in organizational towns, in Tehran/Iran in 2014. Path analysis is a straightforward extension of multiple regression. Its aim is to provide estimates of the magnitude and significance of hypothesized causal connections between sets of variables (Lleras, 2005). In order to calculate sample size of path analysis, for each independent variable, 30 subjects were considered (Nunnally & Bernstein, 1994). In this research, TPB model included 5 Predictor variables and finally 180 women participated. We selected participants with random multi-stage cluster sampling. In this method, we assigned randomly two organizational towns among the list of organizational towns in Tehran. Then we allocated randomly a number of the buildings (consisted of several houses) amongst these towns. Finally, a number of the houses were randomly chosen in selected buildings. The inclusion criteria were: aged between 18 - 64 years old, being literate, wish to participate, not currently being investigated by other researchers, no history of chronic conditions, mental and disabling disorders. The exclusion criteria included to have medical contraindications for exercising. The target behavior of the present study was selected according to WHO recommendations, i.e. Adults aged 18–64 should do at least 150 minutes of moderate-intensity physical activity throughout the week (5 day per week, daily at least 30 minutes) (WHO, 2010).

### 2.2 Measures

Data were gathered by the short form of the International Physical Activity Questionnaire (IPAQ), demographic and TPB scale. The IPAQ has become the most widely used physical activity questionnaire (van Poppel, Chinapaw, Mokkink, Van Mechelen, & Terwee, 2010). The validity and reliability of the IPAQ were approved in several studies (Booth et al., 2003) and current research (intraclass correlation coefficient = 0.85). The short form records the activity of four intensity levels: (a) vigorous-intensity activity; (b) moderate-intensity activity; (c) walking; and (d) sitting (Lee, Macfarlane, Lam, & Stewart, 2011). There are three levels of physical activity proposed to classify populations: low, moderate and high (International Physical Activity Questionnaire, 2005). A trained interviewer collected the IPAQ data. Demographic scale composed of 6 items. In order to construct the TPB scale, we generated an item pool extracted from the TPB literature (Andersson & Moss, 2011; Darker, French, Eves, & Sniehotta, 2010; French et al., 2011; Mok & Lee, 2013; Nelson, Benson, & Jensen, 2010; Poobalan, Aucott, Clarke, & Smith, 2012) especially Ghazanfari et al. study (Ghazanfari, Niknami, Ghofranipour, Hajizadeh, & Montazeri, 2010). Also Ajzen (Ajzen, 2002) and Francis et al. manual (Francis et al., 2004) was utilized in constructing of TPB scale. Banville et al. (Banville, Desrosiers, & Genet-Volet, 2000) method was employed to cross culturally translate of this scale. An expert panel consisted of 10 health and physical education specialists qualitatively evaluated grammar, wording, item allocation and scaling of the TPB questionnaire. In the quantitative phase, we calculated two indicators: the content validity index (CVI) and the content validity ratio (CVR). CVI assesses the relevancy, simplicity and clarity of an item of the content represented in an instrument (Lynn, 1986; Waltz & Bausell, 1981). Polite and Beck recommended 0.80 for the acceptable lower limit of the CVI value (Polit & Beck, 2004). CVR examines the essentiality of an item in an instrument (Lawshe, 1975). In order to assess face validity, 10 women in target group were asked to evaluate the scale and indicate if they felt the difficulty, Irrelevancy or ambiguity in responding to the questionnaire (qualitative method). In the quantitative phase, we calculated the impact score (frequency × importance) to indicate the percentage of women who identified the item was important or quite important. Those items associated with an impact score equal or greater than 1.5 were considered appropriate (Lacasse, Godbout, & Series, 2002). We evaluated the reliability of the TPB scale by means of internal consistency and test–retest reliability methods. The internal consistency with the Cronbach’s alpha coefficient was examined in 30 women. The alpha values of 0.70 or above were considered satisfactory (Cronbach, 1951). We estimated the stability (test–retest reliability) of the TPB instrument by intraclass correlation coefficient (ICC) (Bartko, 1966). Women (n=30) completed the questionnaire twice with a 2-week interval. We specified the construct validity of TPB scale by administering exploratory factor analysis (EFA) and confirmatory factor analysis (CFA). We accomplished EFA utilizing the principal component analysis (PCA) and varimax rotation. To determine the best structure, the eigenvalue greater than one and factor loading equal to or greater than 0.4 were applied (Dixon, 2005). We conducted CFA by means of maximum likelihood estimation. The TPB questionnaire included affective attitude (refers to emotion drives engendered by the prospect of performing a behavior) (French et al., 2005), instrumental attitude (which refers to a more cognitive consideration of the extent to which performing a behavior would be advantageous) (French et al., 2005), subjective norms, perceived behavioral control, intention and behavior. Data were collected by trained health education researchers.

The psychometric evaluation of TPB scale was described in detail in a separate sample of the target women in the other unpublished paper. Therefore, current paper presented methods and results of aforesaid study in order to inform readers, briefly.

### 2.3 Statistics and Data Analysis

In the path analysis stage, we assessed the fitness of TPB model applying many fit indices; chi square () should be non-significant to indicate a good fit (Hu & Bentler, 1999). < 3 (Munro, 2010), Root Mean Square Error of Approximation (RMSEA) ≤ .06, Root Mean Square Residual (RMSR) ≤ .08 (Hu & Bentler, 1999) and Comparative Fit Index (CFI), Goodness of Fit Index (GFI), Adjusted Goodness of Fit Index (AGFI), Normed Fit Index (NFI), Non- Normed Fit Index (NNFI) and Incremental Fit Index (IFI) ≥ .90 (Munro, 2010) show a good fit. We analyzed the data through SPSS16 and LISREL8.8 by utilizing the Cronbach’s alpha coefficient, intraclass correlation coefficient, principal component analysis with varimax rotation, maximum likelihood estimation, covariance and correlation matrices and standardized regression coefficients (Beta).

### 2.4 Ethical Issue

The Ethics Committee of Tarbiat Modares University approved this study. All participants gave their permission by signing an informed consent form. All ethical principles were considered in all phases of the research.

## 3. Results

The results of psychometric assessment of the TPB scale were satisfactory. The mean of CVI, CVR, impact score (for items), alpha Cronbach and ICC (for constructs) of this scale were 0.84, 0.86, 4.64, 0.87 and 0.73, respectively. A six-factor solution emerged as a result of an EFA and explained 76.33% of the variance observed. CFA results confirmed the exploratory six factor structure (=378.68, df=239, =1.58, CFI=0.92, RMSEA=0.06).

A total of 180 women (age mean=31. 25 ± 5.18) participated in the path analysis study. In general, 51.1% of women had academic education and 81.7% were housekeeper. Also, 95% of subjects were inactive or less active and 94.5% placed in the lowest category of physical activity. Demographic characteristics and physical activity rate and level of the participants were shown in Table 1.

**Table 1:** Demographic characteristics and physical activity rate/level of the participants (n=180)

Variable	Mean ± SD	N (%)
**Age (years)**	31.25 ± 5.18	
**Number of children**	2.37 ± 0.77	
**Number of family members**	4.38 ± 0.78	
**Physical activity**		
**Day (s) per week**	1.07 ±1.21	
**Minutes per week**	32.33 ± 3.64	
**Education**		
**Primary**		0 (0)
**Secondary/College**		88 (48.9)
**University**		92 (51.1)
**Education of husband**		
**Primary**		0 (0)
**Secondary/College**		54 (30)
**University**		126 (70)
**Occupation**		
**Housewife**		147 (81.7)
**Employed**		33 (18.3)
**Physical activity**		
**Inactive (0 Minutes per week)**		65 (36.1)
**Less active (<150 Minutes per week)**		106 (58.9)
**Active (≥150 Minutes per week)**		9 (5)
**Physical activity**		
*Low		170 (94.5)
**Moderate**		8 (4.4)
**High**		2 (1.1)

* Low: Those individuals who aren’t meeting criteria for Categories Moderate or High are considered to have a ‘low’ physical activity level.

Moderate: 5 or more days of any combination of walking, moderate-intensity or vigorous intensity activities achieving a minimum total physical activity of at least 600 MET-minutes/week.

High: vigorous-intensity activity on at least 3 days achieving a minimum total physical activity of at least 1500 MET-minutes/week or 7 or more days of any combination of walking, moderate-intensity or vigorous-intensity activities achieving a minimum Total physical activity of at least 3000 MET-minutes/week.

**Figure 1:**
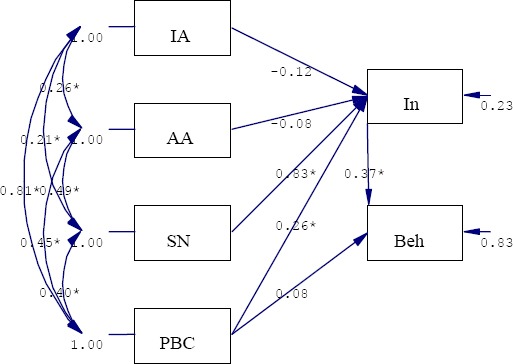
Path diagram of TPB * p<0.01

The findings indicated TPB explained 77% and 17% of the intention and behavior variance (Figure 1). Subjective norms (β=0.83) and behavioral intention (β=0.37) were the strongest predictors of intention and behavior, respectively. The instrumental and affective attitude had no significant path toward dependent variables. Also, the direct path of perceived behavioral control to behavior was non-significant (Table 2). Fit indices displayed that TPB fitted to the data (Table 3).

**Table 2:** Direct, indirect and total effects of TPB structures

Independent Variables	Dependent Variables	Direct Effects	Indirect Effects	Total Effects
**Instrumental Attitude**	Behavioral Intention	-0.12	-	-0.12
	
**Affective Attitude**		-0.08	-	-0.08
	
**Subjective Norms**		0.83*	-	0.83*
	
**Perceived Behavioral Control**		0.26*	-	0.26*

**Instrumental Attitude**	Behavior	-	-0.05	-0.05
	
**Affective Attitude**		-	-0.03	-0.03
	
**Subjective Norms**		-	0.31*	0.31*
	
**Perceived Behavioral Control**		0.08	0.10*	0.18*
	
**Behavioral Intention**		0.37*	-	0.37*

* p<0.01.

**Table 3:** Fit indices of TPB model

χ^2^*	df	χ^2^/df	CFI	GFI	AGFI	NFI	NNFI	IFI	RMSEA	SRMR
7.27	6	1.21	1	0.99	0.95	0.99	0.99	1	0.02	0.03

* p>0.05.

Constructs: IA: Instrumental Attitude, AA: Affective Attitude, SN: Subjective Norms, PBC: Perceived Behavioral Control, In: Intention, Beh: Behavior

## 4. Discussion

This survey was the first (theory based) research to evaluate physical activity behavior and its determinants in military personnel’s wives in Iran. It is of great importance to recognize the relevant and effective factors on physical activity of different groups for designing and evaluating the efficient health education interventions (Robbins, Gretebeck, Kazanis, & Pender, 2006) to increase and sustain exercise behavior and active lifestyle. Despite the lack of early access to exercise behavior statistics of military staff’s spouses, this investigation demonstrated that only 5% of these women were adequately active. Therefore, it was required to examine the exercise behavior in this target group. The TPB scale was confirmed in term of content, face, construct validity and reliability.

The findings of path analysis demonstrated the TPB variables explained 77% and 17% of the variance in exercise intention and behavior, respectively. Hagger (Hagger et al., 2002), McEachan et al. (McEachan et al., 2011) reported the TPB predicted 41-46% of intention variance and 24-36% of behavior variance. Different prediction of intention and behavior by TPB may be due to survey different behavior and target group in current research (Fishbein & Ajzen, 1975). For instance, Ghazanfari study on diabetic women’s physical activity indicated TPB accounted for 15% intention and 11% behavior variance (Ghazanfari, 2010).

Subjective norms and perceived behavioral control (PBC) significantly predicted intention. Blue (Blue, 2007), Omondi et al. (Omondi, Walingo, Mbagaya, & Othuon, 2010) and Didarloo et al. (Didarloo et al., 2011) studies of diabetic patients revealed that subjective norms as an important determinant of exercise intention. However, some researchers noted subjective norms as a weak predictor of intention than attitude and perceived behavioral control (Bozionelos & Bennett, 1999; Tavousi, 2009). As regards PBC, similarly Hosseini et al. found it was positively related to exercise intention and behavior in female students (Hosseini, Khavari, Yaghmaei, & Alavi Majd, 2010). Although, in the present study, PBC had no significant direct path to behavior and indirectly related to behavior through intention. In other word, perceived control of subjects was effective before decision making for exercise behavior. In contrast to this result, PBC had no significant effect to exercise intention of diabetic women in Ghazanfari research, whereas, it directly predicted behavior (Ghazanfari, 2010). Perhaps a feeling of control of physical activity was more important to act (compare to motivate to exercise behavior) in the patients than healthy peoples. Also Estabrooks and Carron concluded control beliefs were good predictors of exercise attendance in the elderly (Estabrooks & Carron, 1998).

Behavioral intention was the most powerful determinant of behavior. Subjective norms and PBC indirectly explained exercise behavior, respectively. Also, Moeini et al. suggested intention and enabling factor significantly predicted physical activity (Barati, 2011). Findings of Hosseini et al. the survey showed subjective norms, PBC and intention correlated to exercise (Hosseini et al., 2010). However, Prapavessis et al. found PBC accounted for 11% exercise behavior variance, but, the intention had no significant path to behavior in cardiac patients (Prapavessis et al., 2005).

The instrumental and affective attitude had no significant path to intention and behavior. However, these structures significantly correlated to subjective norms and PBC. This result was supported by Prapavessis et al. research (Prapavessis et al., 2005). But Hagger et al. reported attitude was the strongest predictor of physical activity intention in the most studies based on the Theory of Reasoned Action (TRA) and TPB and mediated intention-behavior relation (Hagger et al., 2002). Similarly, Lippke et al. mentioned attitude as a construct of TPB significantly explained intention of preparation phase people (Lippke, Nigg, & Maddock, 2007). Perhaps perceived social pressure and sense of control over exercise behavior played much more role than attitude in current research participants. For example, Biddle and Nigg suggested PBC and subjective norms are more important than attitude as age rises (Biddle & Nigg, 2000).

Generally, some different results of this research with other studies may be related to assess different health behaviors in individuals with diverse socio-cultural characteristics. As Fishbein and Ajzen noted, is possible to be various relative weights of subjective norms, attitudes and PBC to predict intention and behavior in different communities and people (Fishbein & Ajzen, 1975).

## 5. Conclusion

In sum, this research provides important information about affecting factors to exercise behavior and their relationships, according to TPB in military personnel’s wives. Therefore, it is essential to consider these efficient determinants when designing health education interventions to promote physical activity behavior in this target group. For example, in order to increase exercise intention, health educators should first focus on subjective norms then intervene in PBC. These constructs were promoted by educational methods such as role playing, panel discussion, discussion about behavior facilitator/barriers and etc. Consequently, strengthening of intention leads to desired physical activity behavior.

Finally, it should be noted that present study has some limitations. First, a cross-sectional design was used to describe the relationship between variables. The main characteristic of cross-sectional design is that all data are collected at one time period, thereby limiting the ability to identify cause-and-effect relationships between variables. Second, the data for this study were collected using self-reported questionnaires (TPB scale and IPAQ). Participants may underestimate or overestimate their exercise behavior in self-reported measures (than objective scales), which may have affected the study findings. However, the results of two scales were similar to measure physical activity. The third, the very low levels of physical activity in this research may affect the statistics findings. Fourth, the TPB has been critiqued for focusing on cognitive elements and ignoring the role of another factor (emotion, personality, culture and demographic variables) in behavioral change (Raingruber, 2014; Sharma & Romas, 2011). Hence, perhaps other determinants influenced on forming behavior besides the TPB variables in the present research (TPB). However, it has proven to be an effective model for predicting health- related behavior (Ajzen, 2014; Raingruber, 2014). Fifth, the study of military personnel’s wives who resided in the organizational town in Tehran may limit generalization of the findings to the entire military personnel’s wives in Iran. Therefore, the results of this study have to be interpreted with some caution. Further studies are now needed with regard to larger samples, including military staff’s wives residing in the other areas. Also, it is suggested to evaluate the effect of an educational intervention based on TPB in this target group by applying the findings of the present study.
